# Haematology and plasma biochemistry reference intervals of Española, San Cristobal and Eastern Santa Cruz Galapagos tortoise species

**DOI:** 10.1093/conphys/coae055

**Published:** 2024-08-15

**Authors:** Ainoa Nieto-Claudín, Jamie L Palmer, Maris Brenn-White, Fernando Esperón, Sharon L Deem

**Affiliations:** Charles Darwin Foundation, Charles Darwin Avenue, Santa Cruz 200350, Galapagos Islands, Ecuador; Saint Louis Zoo Institute for Conservation Medicine, One Government Drive, Saint Louis, MO 63110, USA; Saint Louis Zoo Institute for Conservation Medicine, One Government Drive, Saint Louis, MO 63110, USA; Saint Louis Zoo Institute for Conservation Medicine, One Government Drive, Saint Louis, MO 63110, USA; INIA-CISA, Algete-El Casar Road, Valdeolmos 28130, Spain; Veterinary Department, School of Biomedical and Health Sciences, Universidad Europea de Madrid, 28670 Villaviciosa de Odón, Madrid, Spain; Charles Darwin Foundation, Charles Darwin Avenue, Santa Cruz 200350, Galapagos Islands, Ecuador; Saint Louis Zoo Institute for Conservation Medicine, One Government Drive, Saint Louis, MO 63110, USA

**Keywords:** Blood parameters, Chelonians, Chelonoidis spp, reference intervals wildlife health surveillance

## Abstract

Normal reference intervals (RI) of hematologic and biochemical parameters are important for assessing and monitoring the health status of captive and free-living chelonians; however, such information is not available for most wildlife species. Giant Galapagos tortoises are one of the most iconic animals on earth and health information can make an important contribution to their conservation and management. This study provides formal RI of haematology and plasma biochemistry parameters and describes cell morphology along with morphometrics of free-living Eastern Santa Cruz (*Chelonoidis donfaustoi*), Española (*Chelonoidis hoodensis*) and San Cristóbal tortoises (*Chelonoidis chathamensis*). We explored differences in blood parameters between sexes, across the tortoise species in this study and with previously published RI of the Western Santa Cruz tortoise (*Chelonoidis porteri*). Biochemistry parameters of both Santa Cruz species were overall more similar to each other than to Española and San Cristobal tortoises. This research constitutes the first RI for these three Galapagos tortoise species and may be of value for advising captive-breeding and conservation plans. We recommend further research to establish RI in additional tortoise species so we may better understand and interpret haematology and biochemistry parameters as a valuable conservation tool for species of this critically endangered taxon.

## Teaser

We report the first hematology and plasma biochemistry reference intervals for the free-living Galapagos tortoise species of Eastern Santa Cruz, San Cristóbal, and Española Islands. This information may be used to inform diagnostic and management decisions for giant tortoises both in human care and in the wild.

## Introduction

Testudines (turtles and tortoises) are amongst the most threatened vertebrate groups on earth with >60% of their species endangered or already extinct (Lovich *et al.*, 2018). Habitat loss and degradation, human use for food and as pets, climate change and diseases are main threats to turtle survival (Stanford *et al.*, 2020). Galapagos tortoises are the largest extant terrestrial reptiles and amongst the most iconic and long-lived species, yet their health status remains poorly understood (Nieto-Claudín *et al.*, 2021a, 2021b, 2022). The taxonomy of Galapagos tortoises has undergone a number of major shifts and revisions over the past several decades (Garrick *et al.*, 2012, 2015; Poulakakis *et al.*, 2015, 2020) but currently up to 16 species of these often migratory mega-herbivores have been described (Jensen *et al.*, 2022) and at least 12 species are extant in the archipelago (IUCN, 2020). Massive over-harvesting from the 1800s followed by increasing pressure from invasive species and the expansion of the human population caused the extinction of at least two species and dramatic declines in most other species (MacFarland *et al.*, 1974; Conrad and Gibbs, 2021). Captive-breeding programmes carried out by the Galapagos National Park Directorate and the Charles Darwin Foundation have successfully recovered some of the species from the brink of extinction (Cayot, 2021a, 2021b); however, current threats related to climate change, invasive species, habitat degradation, disease, trauma, antimicrobial resistance and illegal trade persist (Ellis-Soto *et al.*, 2017; Blake *et al.*, 2021; Nieto-Claudín *et al.*, 2021b; Nieto-Claudín *et al.*, 2022; Deem *et al.*, 2023).

Hematologic and biochemistry analyses are easy diagnostic and prognostic tools for chelonians. Several diseases (e.g. hemoparasites, inflammatory and infectious diseases) are associated with changes in these blood parameters and therefore, haematology and biochemistry values can be used to diagnose chelonian diseases, to assess the health status of individuals and as a prognostic indicator after treatment (Zhang *et al.*, 2011; Heatley and Russell, 2019). Normal reference intervals (RI) of hematologic and biochemical parameters are considered important for assessing and monitoring the health status of captive and free-living chelonians; however, such evaluations are dependent on reliable reference values for healthy animals (Moller and Heatley, 2022). In recent years, several studies have established RI of hematologic and biochemistry variables of free-living and captive chelonians (Way Rose and Allender, 2012; Mumm *et al.*, 2019; Brenn-White *et al.*, 2022).

Robust haematology and biochemistry RI were recently described by our research group in free-living Western Santa Cruz giant tortoises (*Chelonoidis porteri*) using the same methodology (Nieto-Claudín *et al.*, 2021a). Additionally, blood parameters on 23 San Cristobal tortoises (*Chelonoidis chathamensis*) kept under human care for a long time were previously described in 2018 by Lewbart *et al.*, 2018 No other efforts have been conducted yet to describe blood values on the other Galapagos tortoise species nor to compare between species, locations or morphotypes.

As an ongoing effort to close this gap and provide accurate and updated data for tortoise captive-breeding and restoration programmes, we calculated biochemical and haematology RI, described cell morphology and collected morphometric measurements and body weight of free-living tortoises from Eastern Santa Cruz (*Chelonoidis donfaustoi*), Española (*Chelonoidis hoodensis*) and San Cristóbal (*Chelonoidis chathamensis*) Islands.

## Materials and Methods

### Study site

**Figure 1:**
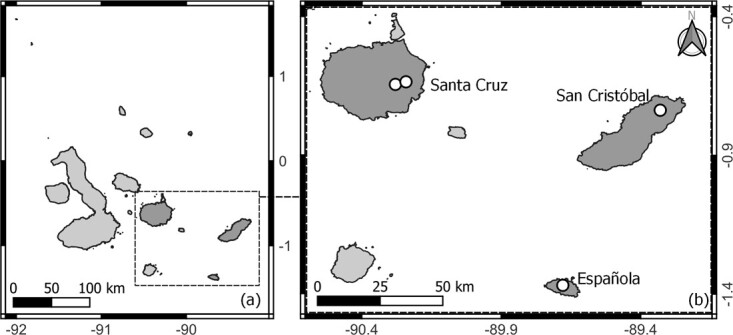
Sampling locations of 51 Eastern Santa Cruz tortoises (*C. donfaustoi*), 45 Española tortoises (*C. hoodensis*) and 40 San Cristobal tortoises (*C. chathamensis*) used to describe haematology and plasma biochemistry reference intervals.

The current study was conducted in three different islands of the archipelago, as part of a long-term health assessment within the Galapagos Tortoise Movement Ecology Programme (GTMEP) and in collaboration with the Galapagos National Park Directorate (GNPD). In San Cristóbal Island, free-living tortoises (*C. chathamensis*) were sampled on a 2-day trip in January 2020. The sampling site was located on the north-east area of the island, near Punta Pitt (S00.73772, W089.33103). Although this particular area is not populated by humans, invasive goats are present and cause significant impacts on the ecosystem. On the most human-populated island, Santa Cruz, Eastern giant tortoises (*C. donfaustoi*) were sampled between 2018 and 2019 along their migration routes, within humid and transition habitat zones and within agricultural (S00.64524, W090.2808) and protected areas (S00.63587, W090.24271). In Española Island, located in the south-east of the archipelago and uninhabited by humans and livestock, *C. hoodensis* tortoises were sampled on a 5-day field trip conducted in July 2019 (S01.3695, W089.67966) (Fig. 1). In both Eastern Santa Cruz and Española Islands, tortoises that have been under long-term monitoring via GPS devices (Blake *et al.*, 2021) were also sampled at the same time and are included for RI calculations within the total sample size.

### Sampling design and collection

For all individuals we recorded morphometric measurements (curved carapace and plastron length and width) using a flexible 150-cm measuring tape and weighed each tortoise with a hanging scale (precision of ±0.5 kg). We performed a visual examination to assess general health status, determined the sex of mature animals based on tail length and plastron concavity and classified their age (i.e. adults, sub-adults, juveniles) based on curved carapace length (CCL) (Nieto-Claudín *et al.*, 2021a). We collected up to 5 ml of blood from the brachial vein of each tortoise with a 6-ml heparinized syringe and a 20G 1.5-inch needle, and made two blood films immediately at the field site, fixed using high-quality methanol (Fixative 1, JorvetTM Diff Quick Stain Kit, Jorgensen Laboratories, USA), air dried, labelled with tortoise ID and stored in a slide box (as previously described in Sheldon *et al.*, 2016). Up to 3 ml of blood was immediately transferred to a lithium heparin tube to avoid clotting. We kept heparinized blood at 4^°^C until analysis within the same day. We identified tortoises by microchips previously placed by Galapagos National Park Service rangers. If no microchip was detected, we placed a new subcutaneous microchip (DATAMARS®) in the caudo-ventral area of the left hind leg.

### Haematology and plasma biochemistry

We used heparinized blood samples to perform packed cell volume (PCV) and total solids (TS) within 8 h of sample collection. PCV was determined using high-speed centrifugation of blood-filled microhematocrit tubes, and manual TS plasma analysis was conducted with a clinical refractometer (J-351, Jorgensen Laboratories, USA). Plasma was immediately separated by high-speed centrifugation and kept frozen at −20°C until biochemistry was performed within 2 weeks of collection.

We used modified Wright Giemsa stain (JorvetTM Diff Quick Stain Kit, Jorgensen Laboratories, USA) to stain blood films following manufacturer’s instructions. We determined the estimated total white blood cell (WBC) counts using the highest quality blood film from each individual. White blood cell counts were read at 40× magnification on 10 different fields of the monolayer and averaged using the following equation: WBC (× 10^9^ cells/l) = (AVG 10 field on 40×) × 1.6 (Sheldon *et al.*, 2016; Nieto-Claudín *et al.*, 2021a). We performed WBC differential counts by examining 100 WBCs on the same blood film at 100× magnification. We calculated estimated values (%) for heterophils, lymphocytes, monocytes, eosinophils and basophils. We determined heterophil:lymphocyte ratio (H:L) from the differential. Absolute values (ABS) for each white cell morphotype were calculated as follows: ABS WBC Diff (× 10^9^ cells/l) = [ABS WBC (× 10^9^ cells/l)] × [WBS Diff (%)] × 0.01.

We performed biochemistry (Avian/Reptilian VetScan® Profile) on thawed frozen plasma. The biochemical panel included albumin (Alb), bile acids (BA), aspartate aminotransferase (AST), calcium (Ca), creatinine kinase (CK), glucose (GLU), potassium (K), sodium (Na), phosphorus (P), globulin (Glob), total protein (TP) and uric acid (UA).

We collected all samples under the Galapagos National Park annual research permits PC-35-18, PC-16-19, PC-28-20 and PC-17-21, and the International Animal Care and Use Committee from the Group of Rehabilitation of Endemic Wildlife Species (GREFA-Spain) with registration number 17/001. All samples were processed and analysed at the Charles Darwin Research Station (CDRS).

### Statistical analyses

Reference Value Advisor (RefVal) v.2.1 was used to perform descriptive statistics (mean, median, SD, min and max) and compute 95% RI and 90% confidence intervals (CI) for each analyte following ASCVP recommendations for the determination of reference intervals in veterinary species (Flatland *et al.*, 2013). According to the ASCVP, non-parametric or robust methods are recommended when 40 ≤ × ≤ 120 reference samples are available. Although the robust method performs best when reference data have a symmetrical distribution (with or without transformation), it can be used in the absence of Gaussianity. When 20 ≤ × < 40 reference samples are available, RI should be calculated by robust (distribution independent) or parametric (if Gaussianity can be established) methods (Flatland *et al.*, 2013). Therefore, we used non-parametric methods for analytes with sample sizes ≥40 and robust or parametric methods (Box-Cox transformed when possible) for sample sizes <40.

Symmetry and distribution of data were assessed by the Anderson–Darling test and histogram evaluation. Normality tests are designed for large populations; however, in smaller population sizes it has been demonstrated that a threshold *P*-value of 0.3, instead of 0.05, more accurately characterizes the results (Flatland *et al.*, 2013). For this reason, if the test’s *P*-value was <0.3, the analyte was judged as having a non-Gaussian distribution. We identified outlier values by RefVal using Dixon and Tukey’s range tests. We closely analysed every suspected outlier and manually removed only those attributed to poor sample quality or analytic error.

We assessed the normality of all parameters using the Kolmogorov–Smirnov test. We compared biochemistry and haematology parameters between sexes within each species using Mann–Whitney *U* Test (Wilcoxon Rank Sum Test). We also compared all parameters between the three species of study (*C. hoodensis, C. donfaustoi* and *C. chathamensis*) and with those of the Western Santa Cruz species (*C. porteri*) previously published by Nieto-Claudín *et al.* (2021a), using the Kruskal–Wallis (K–W) test with Bonferroni *post hoc* adjustment. We performed the analyses in R 4.0.2 and IBM® SPSS Statistics 25 and used *P* < 0.05 for all tests other than Anderson-Darling as noted above.

## Results

A total of 136 adult free-living tortoises from three species were encompassed in the current study (including 19 long-term GPS-tracked individuals). The number of animals by location and sex, and the average morphometric measurements, are summarized in Table 1. All individuals were determined to be in good health based on physical evaluations and the lack of visual signs of disease such as acute traumatic wounds or depressed mentation (Nieto-Claudín *et al.*, 2021a).

**Table 1:** Number of individuals and morphometrics (CCL and weight) of tortoises included in the description of Galapagos tortoise Reference Intervals grouped by location, species and sex.

Island	Species	*n* (total)	Sex	*n*	CCL (cm) (min; max)	Weight (kg) (min; max)
Santa Cruz	*C. donfaustoi*	51	Females	24	95.3 (70.6; 116.0)	77.1 (31.0; 125.0)
			Males	27	120.9 (73.0; 148.4)	138.9 (33.5; 205.0)^a^
San Cristóbal	*C. chathamensis*	40	Females	20	66.4 (49.3; 79.4)	24.6 (10.5; 39.5)
			Males	19	79.1 (57.7; 95.2)	37.0 (15.5; 67.0)
			Unknown	1	41.2	6.5
Española	*C. hoodensis*	45	Females	32	63.0 (50.9; 76.4)	27.7 (14.5; 46.0)
			Males	13	84.8 (77.6; 91)	53.5 (40.0; 74.0)

^a^Males >205 kg were not weighted

We described RI and 90% CI of haematology and plasma chemistry parameters for a maximum of 48 Eastern Santa Cruz tortoises (Table 2), 37 San Cristóbal tortoises (Table 3) and 43 Española tortoises (Table 4).

**Table 2:** Reference intervals (RI) and 90% confident intervals (CI) of haematology and plasma biochemistry parameters for Eastern Santa Cruz adult tortoises (*C. donfaustoi*).

**Analyte**	**SI Units**	***n***	**Mean**	**SD**	**Median**	**Min**	**Max**	***P*-value**	**Distribution**	**Method**	**LRL of RI**	**URL of RI**	**LRL CI 90%**	**URL CI 90%**	**Outliers**
PCV	l/l	48	20.1	3.1	20	14	33	0.003	NG	NP	14	31	14.0–16.0	23.8–33.0	2
TS/TP	g/l	48	6.2	1.4	6	3.5	9	0.099	NG	NP	3.6	9	3.5–4.2	8.5–9.0	2
WBC conc.	10^9^/l	40	9.5	2.7	9.2	4.8	16.8	0.683	G	NP	4.8	16.8	4.8–5.8	13.6–16.6	0
Heterophil	%	40	22.3	7.6	21.5	8	39	0.303	G	NP	8.1	39	8.0–14.0	35.9–39.0	0
Heterophil	10^9^/l	40	2.1	1	2	0.46	4.7	0.006	NG	NP	0.5	4.7	0.5–1.0	4.3–4.7	0
Lymphocyte	%	40	63.9	8.3	65.5	45	79	0.165	NG	NP	45.1	78.9	45.0–53.0	74.0–79.0	0
Lymphocyte	10^9^/l	40	6.1	2	6	2.3	10.7	0.299	NG	NP	2.3	10.7	2.3–3.6	9.4–10.7	0
Monocyte	%	40	4.4	2.9	4	0	13	0.064	NG	NP	0	13	0–0	9.0–13.0	0
Monocyte	10^9^/l	40	0.4	0.3	0.4	0	1.4	0.045	NG	NP	0	1.4	0–0	0.9–1.4	0
Eosinophil	%	40	3.2	1.9	3	0	8	0.008	NG	NP	0	8	0.0–1.0	6.0–8.0	0
Eosinophil	10^9^/l	40	0.3	0.2	0.3	0	0.6	0.009	NG	NP	0	0.1	0.0–0.1	0.6–0.7	0
Basophil	%	40	6.3	3.6	6	0	16	0.196	NG	NP	0	15.9	0.0–0.1	12.0–16.0	0
Basophil	10^9^/l	40	0.6	0.3	0.6	0	1.3	0.428	G	NP	0	1.3	0.0–0.2	1.1–1.3	0
H:L	%	40	0.4	0.2	0.3	0.12	0.9	0.028	NG	NP	0.1	0.9	0.1–0.2	0.7–0.9	0
Sodium	mmol/l	47	132.1	4.5	132	124	141	0.363	G	NP	124.2	141	124.0–126.0	130.0–141.0	1
Potassium	mmol/l	47	7.4	0.8	7.4	5.7	8.6	0.154	NG	NP	5.8	8.6	5.7–6.1	8.6–8.6	1
Calcium	mmol/l	44	13.1	3.4	11.9	8	20.1	0.000	NG	NP	8.1	20.1	8.0–9.7	20.1–20.1	0
Phosphorus	mmol/l	45	4.1	0.7	4.2	2.3	5.4	0.557	G	NP	2.4	5.4	2.3–3.2	5.2–5.4	3
Uric acid	μmol/l	48	1.7	0.7	1.6	0.4	3.2	0.334	G	NP	0.4	3.2	0.4–0.7	2.9–3.2	0
AST	U/l	44	51.9	14.4	49.5	23	93	0.084	NG	NP	24.3	91.8	23.0–33.8	76.4–93.0	4
CK	U/l	39	1455.5	907.4	1262.0	292	3957	0.003	NG	RT	271.8	3828.3	198.6–399.4	3135.2–4670.7	3
Glucose	mmol/l	48	65.4	24.6	67.5	23	120	0.527	G	NP	23	119.8	23.0–28.0	105.0–120.0	0
Total protein	g/l	48	5.5	1	5.6	2.7	7.7	0.452	G	NP	2.9	7.6	2.7–4.2	7.0–7.7	0
Albumin	g/l	48	1.5	0.5	1.6	0.3	2.4	0.419	G	NP	0.4	2.4	0.3–1.0	2.2–2.4	0
Globulin	g/l	41	4	0.7	4	2.3	5	0.402	G	NP	2.3	5	2.3–3.0	5.0–5.0	1

P, parametric; NP, non-parametric; R, robust; add T, transformed, if data was transformed to Gaussian prior to applying parametric or robust methods. According to ASVCP guidelines, the analyte would be judged as having a non-Gaussian (NG) distribution if the test’s *P*-value <0.3.

**Table 3:** Reference intervals (RI) and 90% confident intervals (CI) of haematology and plasma biochemistry parameters for San Cristobal tortoises (*C. chathamensis*).

**Analyte**	**SI Units**	***n***	**Mean**	**SD**	**Median**	**Min**	**Max**	***P*-value**	**Distribution**	**Method**	**LRL of RI**	**URL of RI**	**LRL CI 90%**	**URL CI 90%**	**Outliers**
PCV	l/l	37	19.5	4.1	20	10	28	0.019	NG	PT	9.6	26.6	5.9–13.0	25.2–28.0	3
TS/TP	g/l	36	5.1	1.1	5	3.2	7.8	0.003	NG	PT	3.4	7.7	3.1–3.7	6.9–8.7	4
WBC conc.	10^9^/l	37	9.6	2.1	9.1	5.28	14.08	0.146	NG	R	4.8	13.9	3.9–5.7	12.6–15.0	0
Heterophil	%	37	20.3	6.6	19	10	37	0.074	NG	RT	10	37.8	8.7–11.5	32.5–44.2	0
Heterophil	10^9^/l	37	1.9	0.7	1.8	0.69	3.52	0.216	NG	RT	0.8	3.7	0.6–0.9	3.2–4.2	0
Lymphocyte	%	37	67.2	7.5	69	45	80	0.013	NG	PT	47.6	78.5	34.6–55.6	76.5–80.5	0
Lymphocyte	10^9^/l	37	6.5	1.8	6.2	3.43	10.7	0.200	NG	RT	3.4	10.8	3.0–4.0	9.5–12.1	0
Monocyte	%	37	5.6	2.3	5	3	10	0.004	NG	PT	2.3	12.1	2.0–2.6	10.5–13.7	0
Monocyte	10^9^/l	37	0.5	0.2	0.5	0.16	1.14	0.126	NG	RT	0.1	1.1	0.1–0.1	1.0–1.3	0
Eosinophil	%	37	0.6	0.9	0	0	3	0.000	NG	P	0	2.4	0–0	1.7–2.9	0
Eosinophil	10^9^/l	37	0.1	0.1	0	0	0.31	0.000	NG	P	0	0.2	0–0	0.1–0.3	0
Basophil	%	37	6.4	3.3	6.0	0	15	0.122	NG	R	0	12.8	0–0	10.8–14.8	0
Basophil	10^9^/l	37	0.6	0.3	0.5	0	1.22	0.329	G	R	0	1.1	0–0	1.1–1.3	0
H:L	%	37	0.3	0.1	0.3	0.12	0.82	0.001	NG	RT	0.1	0.7	0.1–0.2	0.6–0.9	0
Sodium	mmol/l	34	130.3	2.5	131	126	135	0.088	NG	PT	125	135.1	123.5–126.4	134.0–136.2	1
Potassium	mmol/l	36	6.5	1.1	6.4	4.2	8.6	0.178	NG	RT	4.2	8.8	3.6–4.7	8.3–9.4	1
Calcium	mmol/l	36	10.6	3.1	10	5.4	16.1	0.000	NG	RT	5.3	17.3	5.0–6.1	15.6–20.4	0
Phosphorus	mmol/l	36	3.7	0.7	3.8	2.3	5.8	0.406	G	RT	2.3	5.4	2.0–2.7	5.0–5.7	1
Uric acid	μmol/l	36	1.7	0.6	1.5	0.7	3.2	0.045	NG	RT	0.7	3.2	0.6–0.8	2.7–3.7	1
AST	U/l	33	36.2	13	33	20	73	0.000	NG	RT	20.5	70.8	19.4–22.2	57–88.9	4
CK	U/l	26	1992.5	899.7	1940.5	191	3616	0.965	G	RT	221.4	3999.9	0.0–674.3	3439–4497.5	6
Glucose	mmol/l	37	43.9	15.9	39	22	87	0.000	NG	RT	25.1	98.2	23.2–27.3	68.4–151.7	0
Total protein	g/l	35	4.5	0.8	4.6	2.8	5.7	0.022	NG	RT	2.5	5.8	1.4–3.3	5.6–6.0	2
Albumin	g/l	31	1.5	0.3	1.5	1	2.1	0.228	NG	PT	1	2.2	0.9–1.1	2.0–2.4	1
Globulin	g/l	32	3.2	0.4	3.3	2.5	4.2	0.459	G	PT	2.4	4.1	2.3–2.6	3.9–4.4	0

P, parametric; NP, non-parametric; R, robust; add T, transformed, if data was transformed to Gaussian prior to applying parametric or robust methods. According to ASVCP guidelines, the analyte would be judged as having an NG distribution if the test’s *P*-value <0.3.

**Table 4:** Reference intervals (RI) and 90% confident intervals (CI) of haematology and plasma biochemistry parameters for Española tortoises (*C. hoodensis*).

**Analyte**	**SI Units**	***n***	**Mean**	**SD**	**Median**	**Min**	**Max**	***P*-value**	**Distribution**	**Method**	**LRL of RI**	**URL of RI**	**LRL CI 90%**	**URL CI 90%**	**Outliers**
PCV	l/l	43	21.3	3.4	21	15	30	0.523	G	NP	15.1	29.8	15.0–16.1	26.7–30.0	2
TS/TP	g/l	42	5.3	0.8	5.2	3.9	6.8	0.504	G	NP	3.9	6.8	3.9–4.0	6.7–6.8	2
WBC conc.	10^9^/l	26	8.3	4.1	7.9	2.56	17.12	0.134	NG	RT	2.1	18.7	0.0–2.9	15.1–22.2	0
Heterophil	%	25	14.6	5.8	12	7	26	0.093	NG	PT	3.7	28	1.2–6.5	23.8–32.7	1
Heterophil	10^9^/l	25	1.1	0.6	1.2	0.26	2.57	0.692	G	RT	0.2	2.4	0.0–0.4	2.0–2.9	1
Lymphocyte	%	25	74.4	8.8	77	58	89	0.284	NG	RT	51.1	90.7	43.8–60.9	87.4–93.6	1
Lymphocyte	10^9^/l	25	6.3	3.6	5.7	1.67	14.55	0.045	NG	RT	1.4	17.7	1.0–2.1	13.2–22.6	1
Monocyte	%	25	2.7	2.0	3	0	7	0.138	NG	R	0	6.9	0–0	5.0.5–8	1
Monocyte	10^9^/l	25	0.2	0.2	0.1	0	0.62	0.147	NG	PT	0	0.6	0–0	0.5–0.8	1
Eosinophil	%	25	0.5	0.7	0	0	2	0.000	NG	P	0	1.9	0–0	1.3–2.3	1
Eosinophil	10^9^/l	25	0	0	0	0	0.18	0.000	NG	P	0	0.1	0–0	0.1–0.2	1
Basophil	%	25	7.7	4.2	7.0	0	15	0.694	G	R	0	16.6	0.0–1.2	13.7–18.7	1
Basophil	10^9^/l	25	0.6	0.5	0.5	0	1.74	0.070	NG	RT	0	2	0.0–0.1	1.5–2.6	1
H:L	%	25	0.2	0.1	0.2	0.08	0.45	0.029	NG	PT	0.1	0.5	0.0–0.1	0.4–0.7	1
Sodium	mmol/l	37	132.4	5	132	113	142	0.008	NG	RT	120.1	140.5	116.5–124.3	138.4–143.0	1
Potassium	mmol/l	36	7	1	6.9	4.6	8.6	0.082	NG	RT	4.7	9.1	4.1–5.2	8.5–9.5	2
Calcium	mmol/l	39	10.3	2.5	9.8	5.8	16.1	0.012	NG	R	4.5	14.7	3.6–5.8	13.6–16.3	0
Phosphorus	mmol/l	38	3.5	0.7	3.5	1.7	5.3	0.114	NG	RT	2.1	4.9	1.6–2.4	4.5–5.3	1
Uric acid	μmol/l	39	1.7	0.5	1.8	0.7	2.8	0.077	NG	PT	0.8	2.6	0.5–1	2.4–2.8	0
AST	U/l	36	66.7	20.5	67	33	122	0.856	G	RT	30.8	114.4	25.7–37.9	102.4–128.1	1
CK	U/l	28	2911.4	1810.7	2716.5	373	7804	0.378	G	RT	298.4	7675	0.0–691.4	6165.2–9521.1	0
Glucose	mmol/l	39	46.4	12.9	47	22	84	0.502	G	RT	23.1	76.1	18.8–28.7	68.3–84.2	0
Total protein	g/l	39	4.8	0.6	4.8	3.4	5.8	0.670	G	RT	3.4	5.9	3.0–3.8	5.7–6.1	0
Albumin	g/l	37	1.7	0.3	1.7	1.2	2.3	0.291	NG	PT	1.1	2.3	1.0–1.3	2.1–2.4	1
Globulin	g/l	37	3.1	0.4	3.1	2.2	3.9	0.119	NG	RT	2.2	4	2.0–2.4	3.8–4.2	0

P, parametric; NP, non-parametric; R, robust; add T, transformed, if data was transformed to Gaussian prior to applying parametric or robust methods. According to ASVCP guidelines, the analyte would be judged as having an NG distribution if the test’s *P*-value <0.3.

Comparisons between sexes showed significant differences in Eastern Santa Cruz tortoises, with females having lower PCV and GLU than males, and higher TS, Ca, P and Alb (*P* < 0.001). In Española tortoises, female samples were significantly more lipemic and showed lower values of PCV, TP, Glob, K and Na values when compared to males (*P* < 0.001). No significant differences between sexes were observed in San Cristobal tortoises.

Comparison of haematology parameters between these species (Eastern Santa Cruz, Española and San Cristobal) and results previously published on Western Santa Cruz tortoises (Nieto-Claudín *et al.*, 2021a) showed statistical differences for all analytes but PCV. Both species of Santa Cruz presented significantly higher values of TS when compared to Española and San Cristobal (*P* < 0.001). Western Santa Cruz tortoises presented significantly lower values of WBC, heterophils and basophils, and higher values of lymphocytes and monocytes than the other three species (*P* < 0.001). Overall, Eastern Santa Cruz and San Cristobal tortoises presented similar values for all cell types except eosinophils, with higher numbers found for Eastern Santa Cruz when compared to the other species (*P* < 0.001). The average ratio of H:L was <0.5 for all species, but higher values were found in Eastern Santa Cruz and San Cristobal tortoises (*P* = 0.003), followed by Española (*P* = 0.008) and Western Santa Cruz (*P* < 0.001).

Comparison of biochemistry parameters between the four species showed statistically significant differences amongst several analytes, with both species of Santa Cruz being overall more similar to each other than to those of Española and San Cristobal. Values for TP, Glu, Ca, Phos, Glob and K were significantly higher in both Santa Cruz tortoise species when compared to Española and San Cristobal species. Española tortoise samples showed significantly higher values of AST (*P* < 0.001) than the other species, and San Cristobal had significantly lower AST (*P* < 0.001) compared to the other three species. CK was higher in Española tortoises but only statistically significant when compared to Eastern Santa Cruz tortoises (*P* < 0.001). There were no differences in AST and CK between the two Santa Cruz species. The lowest values of Na were found in Western Santa Cruz tortoises, and this difference was significant when compared to the other three species (*P* < 0.001). There were no differences in UA and Alb values between species. More hemolysis and lipemia were observed in Española samples, although differences were not significant when compared to the other three species.

**Figure 2:**
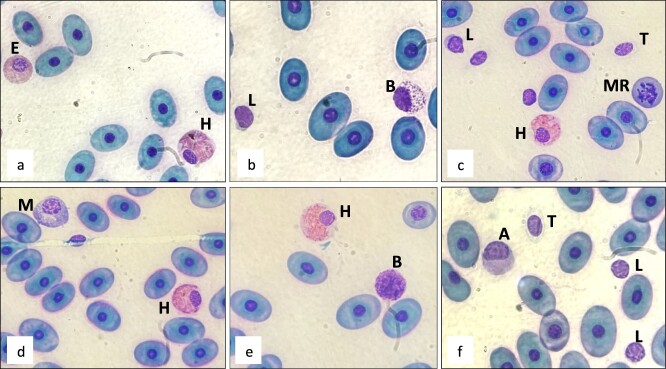
Modified Wright–Giemsa-stained peripheral blood from Española (*C. hoodensis*) tortoises showing: L, lymphocyte; T, thrombocyte; H, heterophil; B, basophil; M, monocyte; A, azurophil; E, eosinophil; MR, mitotic rubricyte. Note degranulation of basophil in image b.

**Figure 3:**
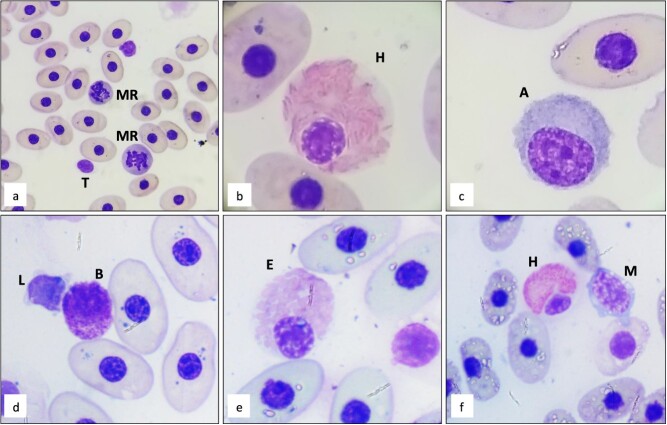
Modified Wright–Giemsa-stained peripheral blood from San Cristobal (*C. chathamensis*) (a–d) and Eastern Santa Cruz tortoises (*C. donfaustoi*) (e–f). L, lymphocyte; T, thrombocyte; H, heterophil; B, basophil; M, monocyte; A, azurophil; E, eosinophil; MR, mitotic rubricyte.

Morphologic characteristics of the WBCs in Española tortoises are provided in Fig. 2 and some examples of San Cristobal and Eastern Santa Cruz in Fig. 3. As is often the case in blood smears made in the field, drying artefacts were present across most slides and evident in erythrocytes. There were no obvious differences identified in WBC morphology between the three species. The predominant WBC morphotype across species was lymphocytes. Mean lymphocyte percentages were 63.9% in Eastern Santa Cruz, 67% in San Cristobal and 74% in Española. Lymphocyte nuclei were eccentric, with dark and clumpy chromatin with little, or scant, light blue to grey cytoplasm. Immature lymphocytes were larger in size with more cytoplasm present. Thrombocytes were differentiated from lymphocytes by their clear cytoplasm and smoother nucleic chromatin. Cells without visible cytoplasm were not scored. The second most abundant WBC morphotype was heterophils. Mean heterophil percentages were 22.3% in Eastern Santa Cruz, 20% in San Cristobal and 14.6% in Española tortoises. Heterophil nuclei were round to oval, off-centred and had dense, clumpy chromatin. In mature heterophils, cytoplasm was pale grey and mostly covered by oval- or rod-shaped, bright pink, glass-like refractive granules. Within the cytoplasm of some heterophils, large grey intracytoplasmic inclusions were visible, making differentiation from eosinophils easier in most cases. Left shift in heterophils was present in all species, with immature heterophils more closely resembling eosinophils; the granules stained more purple, were dull in appearance and were more oval- than rod-shaped. Some of the heterophils, both mature and immature, had basophilic granules, but toxicity was not observed.

Monocytes and azurophils were infrequent and for the differential both were scored as monocytes as recommended for all reptiles except snakes (Stacy *et al.*, 2011). Monocytes were identified by their large round or oval nucleus and light blue cytoplasm often containing vacuoles. Azurophils were similar in size but with more grey cytoplasm, and the nuclei were characteristically oval and eccentric. Combined monocytes and azurophils were low in numbers across species: 4.4% in Eastern Santa Cruz, 5.6% in San Cristobal and 2.7% in Española tortoises. Eosinophils were likewise few in numbers with percentages of 3.2% in Eastern Santa Cruz, 0.6% in San Cristobal and 0.5% in Española tortoises. Eosinophils were round with eccentric nuclei and clear cytoplasm. Granuoles were plump, round and grey to purple or pale pink in appearance. Some eosinophils had cytoplasm with granules very densely packed, which might be confused with young heterophils, but intracytoplasmic inclusions helped make the heterophils stand apart. Basophils were small, dark, compact cells with large nuclei and many dark granules throughout the cytoplasm. The nucleus was often difficult to distinguish from the rest of the cell due to the densely packed granules. In some, degranulation occurred making them slightly more difficult to distinguish as basophils. Mean basophil percentages were 6.3% in Eastern Santa Cruz, 6.4% in San Cristobal and 7.7% in Española tortoises.

Erythrocyte counts were not performed for this study. Mature erythrocytes were oval with very dark, round nuclei and grey to blue cytoplasm. Some basophilic inclusions were observed in the cytoplasma of erythrocytes. Polychromatophils and rubricytes were seen across species but were not found in high numbers in any of the three species. Mitotic rubricytes are noted in Fig. 2 and Fig. 3. No hemoparasites were observed in any slides.

## Discussion

In this work we describe haematology and biochemistry RI parameters of free-living giant tortoises from Eastern Santa Cruz, Española and San Cristobal Islands. To the authors’ knowledge, this is the first publication of formal RI for these Galapagos tortoise species.

A number of factors may explain the differences found in some parameters amongst species: interspecific variations, nutrition, sex, seasonality and population ecology including movement strategies (migrators vs non-migrators) (Deem *et al.*, 2009; Scope *et al.*, 2013; Siest *et al.*, 2013; Blake *et al.*, 2015). The differences in haematology and biochemistry parameters between Eastern Santa Cruz tortoise sexes are in agreement with those described in *C. porteri* (Nieto-Claudín *et al.*, 2021a). Similar to our previous results in *C. porteri*, PCV was slightly higher in males (mean 21.04) than females (mean 19.25), whereas TS, Ca, P and Alb were higher in females most likely due to vitellogenesis. Glucose values were higher in males (mean 79.12) than females (50.48). Blood glucose concentrations in reptiles have been associated with multiple factors including season, sex, diet, temperature and health (Heatley and Russell, 2019). In the current study, most males were sampled within the humid area, whereas females were sampled at different elevations and ecosystems. Migratory studies in Eastern Santa Cruz tortoises have determined that males spend longer periods of time in humid and higher elevations than females (Pike *et al.*, 2021), accessing different feeding resources and being exposed to lower temperatures. Differences between the sexes found in Española tortoise samples might be related to relatively poor sample quality (which may also explain differences in AST and CK values). The uneven distributions of sexes in Española tortoises (32 females and 13 males) may also complicate comparisons with the other species. Seasonality and reproductive status, together with sample size can justify the absence of differences between sexes in San Cristobal tortoises, as all samples were collected during a 2-day period before the breeding season.

The morphology of blood cells in Galapagos tortoises was first documented in *C. porteri* (Nieto-Claudín *et al.*, 2021a). Although little variation in WBC morphology was observed between species, having each documented is useful for future comparison and studies. The only variation in WBC morphology noted between species was related to coloration of the cells, which was likely due to artefact. High humidity during slide preparation (especially in the highlands of Santa Cruz) is likely the cause of drying artefact on the slides. Other factors that may affect cell morphology and slide quality include sample collection technique, lymph dilution, poor slide staining and/or disease (Heatley and Russell, 2019). To avoid some of these factors, slides were always performed by the same person (A.N.C.). Additionally, all slides were prepared animal-side with blood taken directly from a heparinized syringe, minimizing lithium heparin dilution from the blood collection tube. Similar to *C. porteri*, there were no hemoparasites observed in all three species. Given the physical exam and biochemistry findings, neither the presence of immature cells and mitotic figures nor inclusions within some cell types are considered to have pathologic significance.

Some considerations should be taken when interpreting our results. Reference intervals are calculated based on healthy individuals and whilst animals included in the present study were described as clinically healthy based on absence of signs of disease on visual examination, other indicators such as changes in appetite or activity cannot be recorded in free-living species that are sampled once. In addition, biochemistry values were calculated using commercial VetScan avian/reptile rotors that have certain limitations (Nieto-Claudín *et al.*, 2021a; Brenn-White *et al.*, 2022; Palmer *et al.*, 2023). Bile acids could not be calculated in chelonians using these rotors, and other analytes (CK, Alb, Glob) might be affected by sample quality including hemolysis and lipemia (Nieto-Claudín *et al.*, 2021a). Despite these limitations, VetScan is a valuable tool for clinical care and research in chelonians, particularly where there is limited access to reference laboratories (Brenn-White *et al.*, 2022). Seasonality and species-specific ecology should be considered when interpreting blood values. Animals were sampled at different times of the year, which determines reproductive status and movement patterns of these individuals and may influence tortoise physiology, thus blood values (Deem *et al.*, 2009; Blake *et al.*, 2015).

In contrast to haematology, biochemistry parameters in both Santa Cruz species were more similar to each other than to the other species. This can be explained by their distribution and ecology as both occur in an environment with similar food resources and are partially migratory species (Blake *et al.*, 2012). Española and San Cristobal tortoises live on smaller and drier islands where resources are more scarce and uniformly distributed, and migration has not been described to date (Bastille-Rousseau *et al.*, 2017). According to Poulakakis *et al.* (2020), three main genetic clades occur in Galapagos tortoises, with *C. hoodensis*, *C. chathamensis* and *C. donfaustoi* more related to each other than to *C. porteri*. However, blood parameters may be more related to environmental and individual (sex, age, reproductive status) factors than to genetics, explaining why more similarities were found between the two species from Santa Cruz when compared to San Cristobal and Española tortoises.

Globally, there is an increased recognition of the importance of wildlife health to inform conservation actions (Deem *et al.*, 2001), with health assessments being critical for defining the physiological status of a population as well as detecting disease and decline from anthropogenic impacts (Maceda-Veiga *et al.*, 2015; Kophamel *et al.*, 2022; Deem and Holliday, 2023). In Galapagos, blood RI already published have been applied to long-term monitoring of free-living Santa Cruz tortoises as well as in captive-breeding centres and confiscations from illegal trade. However, wildlife health research is still a growing area that requires transdisciplinary teams working together to standardize the methodologies across taxa and greater attention and resources from institutions and international health agencies.

In summary, this study constitutes the first description of reference intervals of haematology and biochemistry parameters for *C. donfautoi, C. chathamensis* and *C. hoodensis,* offering baseline health data for these three endangered and critically endangered Galapagos tortoise species. This information may be used to inform diagnostic and management decisions for giant tortoises both in human care and free-living. Future research on other tortoise species may enhance our understanding and interpretation of chelonian haematology and biochemistry parameters and identify potential new tools that can improve diagnostic ability under field conditions.

## Data Availability

The data underlying this article will be shared on reasonable request to the corresponding author.
